# Dysregulation of microRNAs and their associations with clinical characteristics in recurrent spontaneous abortion patients

**DOI:** 10.1186/s12884-026-09738-2

**Published:** 2026-08-07

**Authors:** Wei Bai, Zhihai Miao, Wanzhong Kong, Zhi Lin, Jin Ye, Xiangyan Chen, Yun Sun, Wenpin Cai

**Affiliations:** 1 Department of Clinical Laboratory, Wenzhou TCM Hospital of Zhejiang Chinese Medical University, NO.9 Jiaowei Road, Wenzhou, Zhejiang 325000 China; 2 Department of Laboratory Medicine, The Second People’s Hospital of Yueqing, Wenzhou, Zhejiang China; 3 Department of Gynecology, Wenzhou TCM Hospital of Zhejiang Chinese Medical University, Wenzhou, Zhejiang China

**Keywords:** Recurrent spontaneous abortion, MicroRNA-182-5p, MicroRNA-2110, MicroRNA-320a-3p, Body mass index

## Abstract

**Background:**

This study aimed to identify microRNA (miRNA) expression profiles associated with recurrent spontaneous abortion (RSA) and to explore whether their dysregulation is linked to relevant clinical characteristics.

**Methods:**

A total of 51 villous specimens were collected from spontaneous abortion (SA) patients, including 31 RSA cases. Three RSA and three control specimens were randomly chosen for miRNA sequencing. Gene Ontology (GO) and Kyoto Encyclopedia of Genes and Genomes (KEGG) analyses were performed to annotate target gene functions and related signaling pathways. Low-depth whole-genome sequencing was performed on all specimens to eliminate confounding effects induced by chromosomal karyotypic abnormalities. RT-qPCR was used to validate the expression of candidate miRNAs.

**Result:**

Thirty-six significantly differentially expressed microRNAs were identified, consisting of 16 upregulated and 20 downregulated miRNAs. The proportion of abnormal chromosomal karyotypes was significantly higher in women with a single spontaneous abortion than in those with two or more spontaneous abortions (*p* < 0.001). The relative expression levels of miR-182-5p, miR-2110, and miR-320a-3p was not significantly different between samples with normal and abnormal karyotype. The expression levels of miR-182-5p and miR-2110 in the villi of the RSA group were significantly lower than those in the control group (0.736 ± 0.139 vs. 1.830 ± 0.480, *p* < 0.01; 1.874 ± 0.476 vs. 2.493 ± 0.582, *p* < 0.05, respectively). No significant difference was observed for miR-320a-3p expression between the RSA and control groups. Similarly, when the primary RSA subgroup was compared with the control group, comparable results were obtained for miR-182-5p, miR-2110, and miR-320a-3p. Notably, the expression level of miR-320a-3p was significantly upregulated in the high BMI group compared with the normal BMI group (*p* < 0.05).

**Conclusion:**

Two major findings emerged from this study. First, miR-182-5p and miR-2110 are associated with RSA, particularly primary RSA. Second, miR-320a-3p appears to indirectly affect reproductive outcomes by participating in obesity-related metabolic pathways, rather than acting as a direct disease-specific marker for RSA. Collectively, these findings suggest that These findings suggest that miR-182-5p and miR-2110 may serve as potential biomarkers for RSA, while miR-320a-3p may reflect obesity-related metabolic status.

## Background

Approximately 15–25% of pregnant women have experienced spontaneous abortion (SA) [1]. However, the exact prevalence of recurrent spontaneous abortion (RSA) is difficult to determine. Most studies report that RSA, defined as two or more pregnancy losses, affects 1–2% of women, with 80% of these losses occurring in early pregnancy [2]. According to the European Society of Human Reproduction and Embryology (ESHRE) criteria, RSA is defined as two or more consecutive pregnancy losses before 24 weeks of gestation [1]. The etiology of RSA is complex, and involves autoimmune dysregulation, endocrine abnormalities, infectious diseases, anatomical factors, and even up to 50% of RSA cases remains unexplained. Some RSA patients have healthy children, but they continue to experience subsequent recurrent pregnancy loss. RSA is one of the most complex and challenging conditions in reproductive medicine, and it is frustrating for patients, their families, and treating physicians, so elucidating the mechanisms underlying of RSA is a pressing issue.

microRNA(miRNAs) is a class of small molecular non-coding RNAs with lengths of approximately 20–22 nucleotides. They play prominent roles in various physiological and pathological biological processes, such as physiological activities, regulating cell proliferation, differentiation, and apoptosis through inhibiting mRNA translation or affecting mRNA degradation [3]. Consequently, microRNAs are closely related to the occurrence and development of diseases including cancer, cardiovascular disease, neurological disease, and pregnancy complications [4]. microRNAs have potential clinical application value in the diagnosis, treatment, and prognosis assessment of diseases [5].

The relationship between microRNAs and RSA has attracted extensive attention and research. Recent studies suggest that the dysregulation of microRNA expression is related to RSA. For example, Jairajpuri et al. [6] identified 10 differentially expressed microRNAs extracted from women with RSA. Among the 10 microRNAs identified, let-7e, miR-221-3p, miR-16, miR-519d, miR-184, and miR-410 were upregulated, whereas miR-21, miR-125, let-7a, and let-7d were downregulated in RSA cases compared with those in control women. MicroRNAs might regulate the occurrence and development of RSA by influencing the decidual cytokine microenvironment [7], inhibiting proliferation and migration of trophoblast [8], and affecting placental angiogenesis. In addition, specific microRNAs have been investigated to differentiate healthy versus disease states as biomarkers in the clinic for cardiovascular, neurological, and pulmonary disorders and various types of cancer [9, 10].

Therefore, we hypothesize that miRNAs may serve as biomarkers or play important functional roles in RSA. However, previous studies have predominantly focused on the maternal level, such as maternal serum or decidual tissue [6, 7], while research involving fetal tissues remains relatively limited. In addition, most of these studies have either merely reported dysregulated miRNA expression or investigated in vitro cellular effects. Few have integrated miRNA expression with clinical characteristics, an approach that holds direct clinical utility for risk stratification, diagnosis, or prognosis assessment.

To systematically investigate the correlation between microRNAs and RSA among pregnant women, we aimed to identify differentially expressed microRNAs in villous samples from RSA patients, and explore the potential roles of these microRNAs in the pathogenesis of RSA using bioinformatics analysis. We further analyzed the association between microRNA expression levels and clinical characteristics. This study may provide potential biomarkers for early risk assessment and contribute to improved clinical management of RSA.

## Methods

### Study subjects

The participants were women of childbearing age who were recruited from the high-risk pregnancy inpatient department of the Wenzhou Hospital of Traditional Chinese Medicine from June 2018 to September 2022. Villous tissues were acquired from patients with spontaneous abortion (SA group) in early pregnancy. Among these patients, those who experienced two or more consecutive spontaneous abortions were defined as recurrent spontaneous abortion (RSA group). The RSA group was classified into two groups: primary RSA group, with no prior live birth or stillbirth, and secondary RSA group, with 1 or more births followed by two or more consecutive losses. Exclusion criteria included: (1) immunological disorders (antiphospholipid syndrome, SLE, or other autoimmune diseases) confirmed by specific autoantibody tests (ANA, anti-dsDNA, lupus anticoagulant, anticardiolipin antibodies, anti-β2 glycoprotein I antibodies and other relevant autoantibodies); (2) endocrine abnormalities (overt or subclinical thyroid dysfunction, PCOS, diabetes mellitus, or hyperprolactinemia) defined by standard laboratory thresholds; and (3) other conditions including genital tract abnormalities, hypertension, thrombotic diseases, microbial infections, and parental chromosomal abnormalities.

The control group comprised healthy fertile Chinese women aged 22–41 years who underwent an induced abortion voluntarily, with a previous history of successful pregnancy and no previous miscarriages, ectopic pregnancies, preterm births, or stillbirths, who voluntarily underwent induced abortion for personal reasons. Villous tissues were collected from induced abortions performed before 12 weeks of gestation and were immediately snap-frozen in liquid nitrogen overnight, and subsequently stored at -80 °C.

The Ethics Committee of Wenzhou Hospital of Traditional Chinese Medicine (Zhejiang, China) approved this study (approval no. WTCM‑H‑2018038). Written informed consent was obtained from each participant.

### microRNA-seq and gene function analysis

For the discovery phase, samples from each group (RSA and control) were randomly selected for microRNA sequencing (miRNA-seq), which was performed by Gysai Biotechnology (Guangzhou, China). Total RNA was extracted using TRIzol reagent (Invitrogen, Carlsbad, CA, USA). Small RNA libraries were constructed using the NEBNext^®^ Multiplex Small RNA Library Prep Set for Illumina^®^ (New England Biolabs, Ipswich, MA, USA) following the manufacturer’s instructions. The libraries were size-selected (140–160 bp) on a 6% polyacrylamide gel (Novex^®^, Thermo Fisher Scientific, Waltham, MA, USA), quantified using the Qubit^®^ dsDNA HS Assay Kit (Thermo Fisher Scientific), and assessed on an Agilent 2100 Bioanalyzer. Pooled libraries were sequenced on an Illumina NovaSeq 6000 platform (Illumina, San Diego, CA, USA) with 150 bp single-end reads by Gysai (Guangzhou, China).

For microRNA expression analysis, raw reads were aligned to the reference genome (GRCh38/hg38) using STAR (v2.7.0), and miRdeep2 (v2.0.1) was used to estimate RNA expression. Normalization was performed using the TMM method implemented in edgeR (v3.36.0). Gene Ontology (GO) enrichment analysis was performed using GOseq (v1.46.0), and Kyoto Encyclopedia of Genes and Genomes (KEGG) pathway enrichment analysis was conducted using the KOBAS (v3.0).

### Chromosomal copy number variation determination

Chromosomal copy number variations (CNVs) number was determined using whole-genome low-depth sequencing. The genomic DNA of the case group was fragmented by random enzymatic digestion and then we constructed DNA libraries using DNA Library Construction Kit from BioelectronSeq Biotechnology Co., Ltd. Sequencing was performed via the BioelectronSeq 4000 platform at an approximate 1× depth. The raw data was subsequently aligned to the reference genome GRCh37/hg19 and analyzed for CNVs at a resolution of 100 kb. The results were searched in databases and reviewed in the literature, following American College of Medical Genetics and Genomics (ACMG) guidelines.

### Reverse transcription-quantitative polymerase chain reaction (RT-qPCR)

The total RNA in the tissue samples was extracted by RNA-easyIsolation Reagent (Nuovezan, Nanjing, China), and the reagent instructions were followed. Subsequently, the RNA purity and concentration were detected by NanoDropOne spectrophotometer. The stem-loop method was used to detect microRNA. The reverse transcription and PCR primers were synthesized by Shanghai Kamu Biotechnology Co., Ltd., and U6 was used as the endogenous control. The primer sequences were as follows:

hsa-miR-182-5p.

RT: 5’- GTCGTATCCAGTGCAGGGTCCGAGGTATTCGCACTGGATACGACAGTGTG − 3’;

F: 5’- GCGTTTGGCAATGGTAGAACT − 3’;

R: 5’- AGTGCAGGGTCCGAGGTATT − 3’.

hsa-miR-2110.

RT: 5’- GTCGTATCCAGTGCAGGGTCCGAGGTATTCGCACTGGATACGACCACTCA − 3’;

F: 5’-TTGGGGAAACGGCCGC − 3’;

R: 5’-AGTGCAGGGTCCGAGGTATT − 3’.

hsa-miR-320a-3p.

RT: 5’- GTCGTATCCAGTGCAGGGTCCGAGGTATTCGCACTGGATACGACTCGCCC-3’;

F: 5’-GCGAAAAGCTGGGTTGAGA − 3’;

R: 5’-AGTGCAGGGTCCGAGGTATT − 3’.

U6 F: 5’-CTCGCTTCGGCAGCACA-3’; R: 5’-AACGTTCACGAATTTGCGT-3’.

The level of microRNA was monitored by the quantitative reverse transcription kit (Biosharp, Shanghai, China) in accordance with the manufacturer’s instructions. Reactions were performed in triplicate by the ABI PRISM 7500 sequence detection system (Perkin Elmer Applied Biosystems, Foster City, CA) and the relative expression of microRNAs was calculated by the comparative cycle threshold (2^−∆∆Ct^).

### Statistical analysis

Statistical data were analyzed by SPSS 22.0 (IBM Corp., Armonk, NY, USA) and GraphPad Prism 9 software (GraphPad Software, San Diego, California, USA). For comparisons between two independent groups, the independent Student’s t-test was used for normally distributed data, and the Mann–Whitney U test was used for non-normally distributed data. For comparisons among three or more groups, one-way analysis of variance (ANOVA) followed by Tukey‘s post-hoc test was applied for normally distributed data, whereas the Kruskal–Wallis test with Dunn’s post-hoc test and Bonferroni correction was used for non-normally distributed data. Categorical variables were compared using the chi-square (χ²) test or Fisher’s exact test, and pairwise comparisons were performed with Bonferroni correction. The differential screening was performed according to *p* < 0.05.

For miRNA sequencing data, miRNAs with |fold change| > 1.5 and raw *p* < 0.05 were initially considered differentially expressed. To account for multiple comparisons, the raw p‑values were adjusted using the Benjamini–Hochberg false discovery rate (FDR) method. FDR < 0.05 was considered statistically significant for the final list of differentially expressed miRNAs.

## Results

### Characteristics of case and control groups

The characteristics of Case and Control groups were presented in Table 1. The control group contained 19 samples. A total of 51 villus samples were collected in the SA group, including 31 cases in the RSA group (19 RSA-P and 12 RSA-S). In addition to the above specimens, three additional samples were recruited from both the control group and the RSA group for miRNA sequencing. No significant differences were observed in age, gestational weeks, and BMI between the control and SA groups, and similarly, no significant differences were observed between the control and RSA groups. Detailed information is presented in Table 1. 

**Table 1:** Characteristics of case and control groups

Characteristics	Control	SA	RSA	*p* ^a^	*p* ^b^
Number (main assays)	19	51	31(RSA-*P* = 19, RSA-S = 12)		
Number (microRNA-seq)	3		3		
Age (years, mean ± SD)	30.74 ± 5.30	31.33 ± 4.40	31.10 ± 3.82	0.635	0.782
Gestational weeks (mean ± SD)	7.88 ± 1.24	8.82 ± 1.96	8.79 ± 2.08	0.056	0.088
Body Mass Index (BMI)	21.32 ± 2.27	21.80 ± 3.25	22.37 ± 3.42	0.559	0.242
Number of Spontaneous Abortion					
Once	0	20			
Twice	0	23	23		
Three and more	0	8	8		
Number of successful pregnancies	≥ 1	≥ 0	≥ 0		
Number of chromosome karyotypes*					
Normal	19	19	16		
Abnormal	0	30	15		

*SA* Spontaneous abortion, *RSA* Recurrent spontaneous abortion

*p*^a^: Comparison between Control and SA groups using Student’s t-test

*p*^b^: Comparison between Control and RSA groups using Student’s t-test

*Two samples of SA group failed in karyotype analysis

### Differentially expressed microRNAs in the villus tissues

**Fig. 1:**
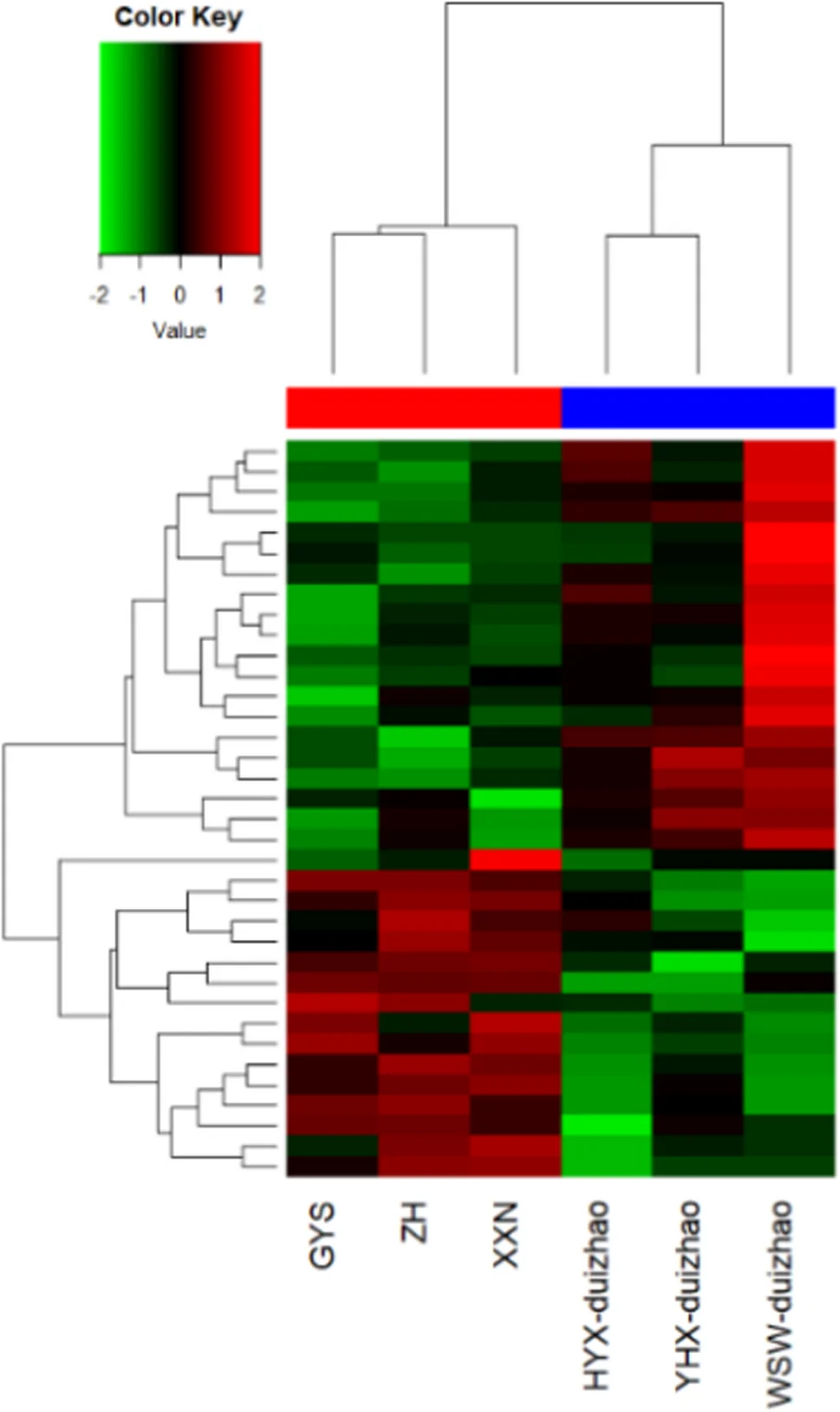
Hierarchical cluster analysis of villous samples to assess the significant expression of microRNA. Red denotes high relative expression and green low relative expression

**Fig. 2:**
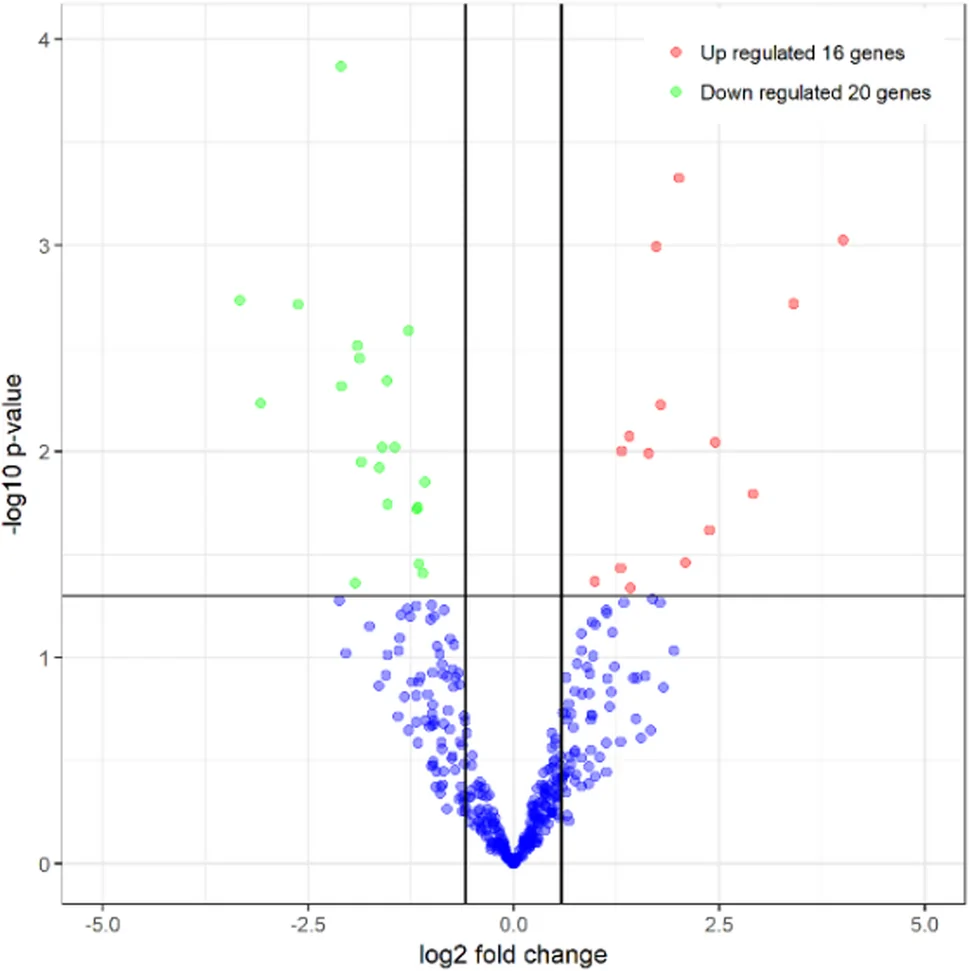
The scatter plot and the volcano plot illustrate the distributions of the data in microRNA profiles. The values of the X-and Y-axes in the scatter plot were the averaged normalized signal values of the group (log2 scaled). The lines in the scatter and volcano plots show a significant fold change. Red dots indicate up-regulation and green dots indicate down-regulation

We profiled the difference in miRNA expression between the RSA group and the control group. Compared with healthy controls, a substantial number of miRNAs were upregulated or downregulated in RSA patients (Fig. 1). Of the 382 detected miRNAs, 36 showed significant differential expression based on a threshold of |fold change| ≥ 1.5 and false discovery rate (FDR) < 0.05, including 16 upregulated and 20 downregulated miRNAs (Fig. 2).

### Gene ontology and kyoto encyclopedia of genes and genomes (KEGG)

**Fig. 3:**
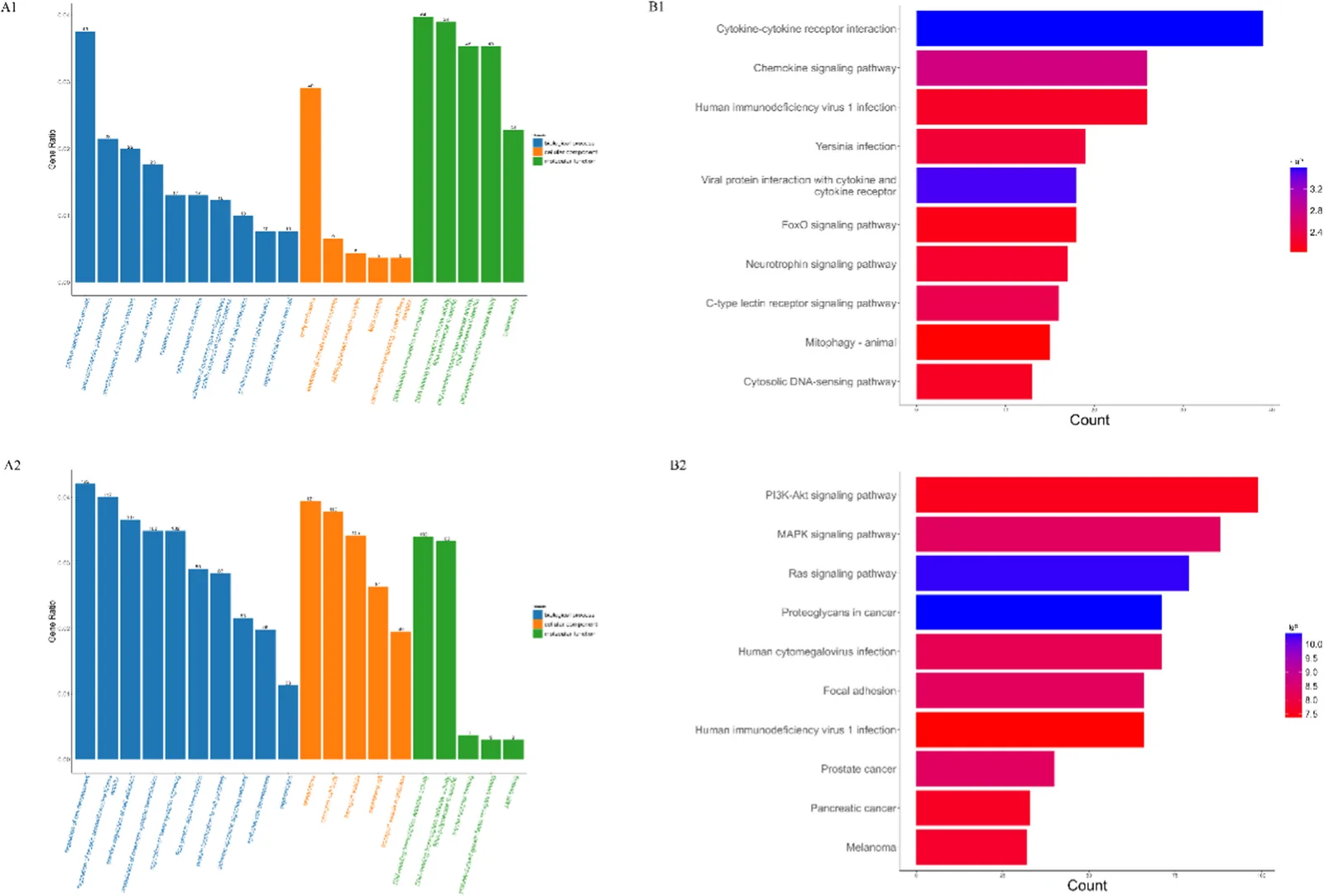
Results of gene enrichment analysis (**A**) GO analysis of microRNA target genes according to biological process, cell component, and molecular function, including analyses of the upregulated (**A**1) and downregulated (**A**2) microRNAs. **B** KEGG analysis of microRNA target genes, including analyses of the upregulated (**B**1) and downregulated (**B**2) microRNAs

GO and KEGG analyses were performed on upregulated and downregulated microRNA target genes, respectively (Fig. 3). The GO analysis results were categorized into three main domains: Biological Process (BP), Cellular Component (CC), and Molecular Function (MF). In BP analysis, pattern specification process, anterior/posterior pattern specification, and morphogenesis of a branching structure were the three most significant processes associated with the upregulated microRNAs, while regulation of cell development, regulation of protein serine/threonine kinase activity and positive regulation of cell adhesion were the three most significant processes associated with the downregulated microRNAs. In CC analysis, early endosome, ionotropic glutamate receptor complex, and AMPA glutamate receptor complex were the three most significant processes associated with the upregulated microRNAs, while presynapse, neuronal cell body, and transport vesicle were the three processes most significantly associated with the downregulated microRNAs. In MF analysis, DNA-binding transcription activator activity, RNA polymerase Ⅱ specific and DNA-binding transcription repressor activity RNA polymerase Ⅱ specific were the three processes most significantly associated with the upregulated microRNAs, while DNA-binding transcription activator activity, RNA polymerase Ⅱ specific and insulin receptor binding were the three most significant enriched processes associated with the downregulated microRNAs. KEGG pathway analysis of related target genes revealed that the top three pathway for up-regulation microRNAs were cytokine-cytokine receptor interaction, chemokine signaling pathway and human immunodeficiency virus 1 infection, while the top three pathway for down-regulation microRNAs were PI3k-Akt, MAPK, Ras signaling pathway.

### The association between relative expression of microRNAs and chromosomal karyotype

**Fig. 4:**
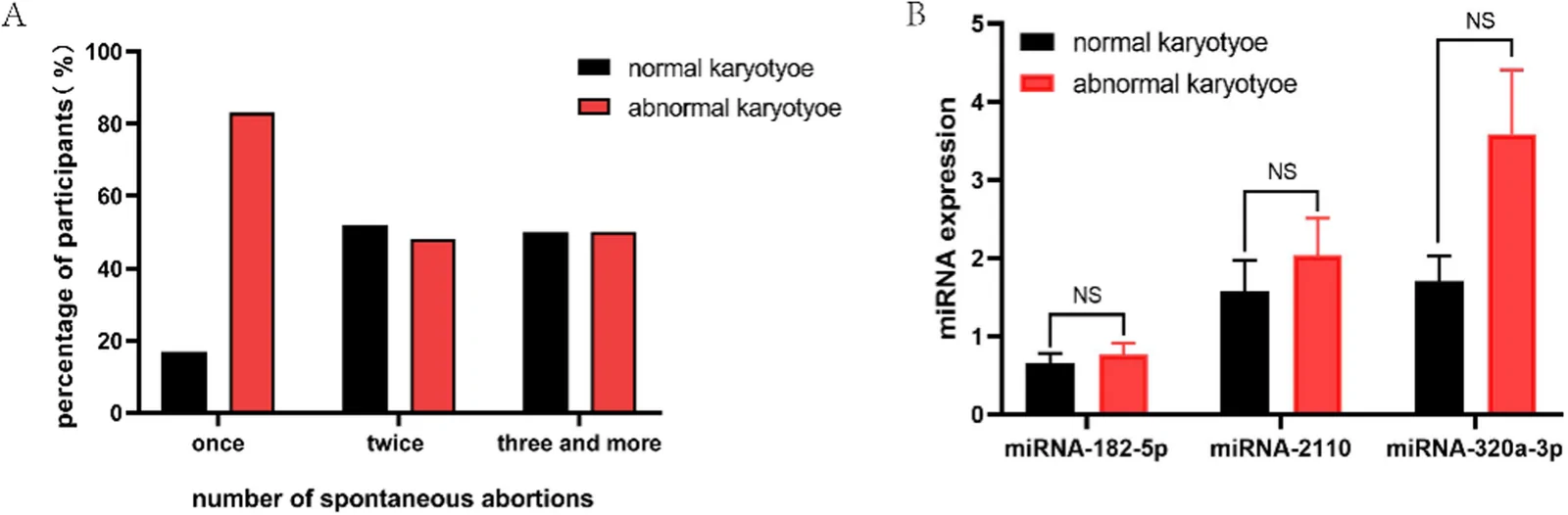
**A** The proportion of normal and abnormal karyotypes in three subgroups according to the number of spontaneous abortions. **B** The relative expression of miR-182-5p, miR-2110, and miR-320a-3p between normal or abnormal karyotypes

Chromosomal karyotypes were investigated in the SA group, which was divided into three subgroups according to the number of spontaneous abortions. The proportion of individuals with normal and abnormal karyotypes differed significantly among the three subgroups (*p* < 0.001), as shown in Fig. 4A. Specifically, the normal karyotype proportions were 17% (3/18), 52% (12/23), and 50% (4/8) for the three subgroups, respectively. The proportion of individuals with abnormal karyotypes was 83% (15/18), 48% (11/23), and 50% (4/8) for the three subgroups, respectively. The proportion of individuals with abnormal karyotypes was significantly greater than that with normal karyotypes in the once subgroup, whereas it was similar in the other two groups. Three microRNAs (miR-182-5p, miR-2110, and miR-320a-3p) were selected for detection, on the basis of references, microRNA databases, bioinformatics analysis, and our previous studies. The relative expression of miR-182-5p, miR-2110, and miR-320a-3p was not significantly different between the normal and abnormal karyotypes (Fig. 4B).

### The relative expression of microRNAs between the RSA and control group

**Fig. 5:**
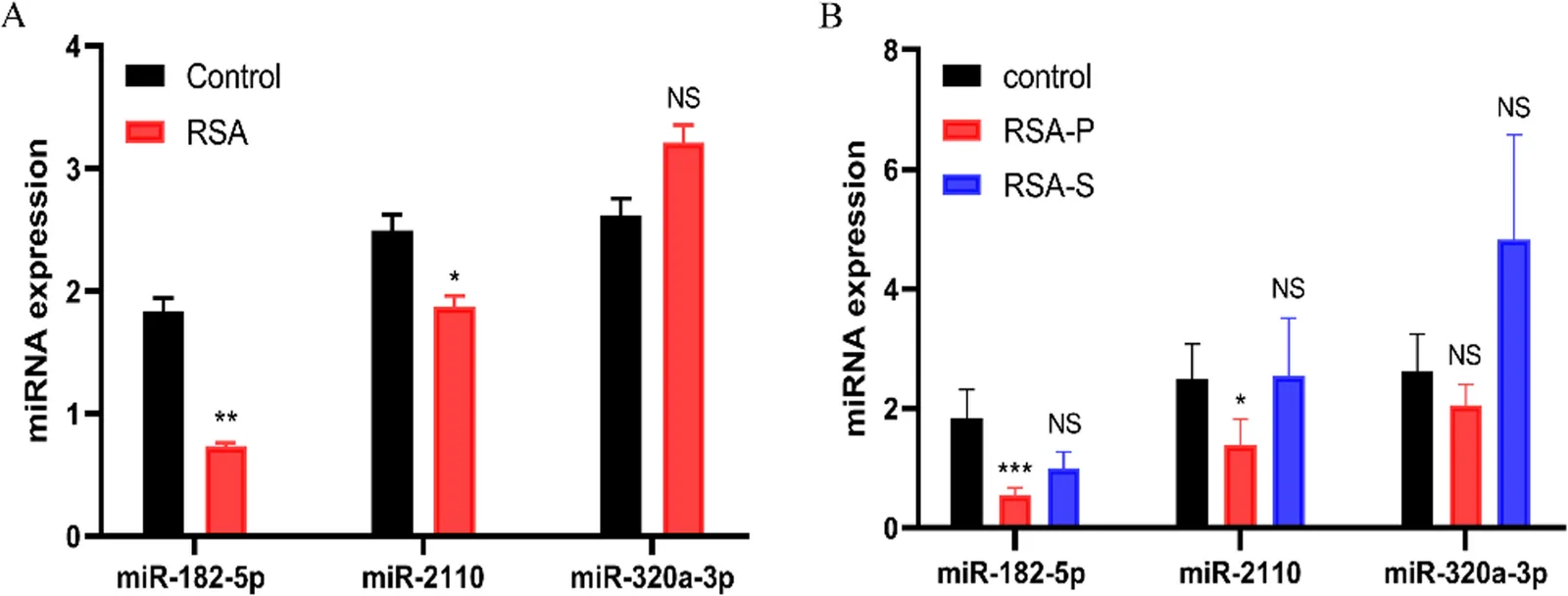
**A** The identified microRNAs expression levels in RSA group and control group. **B** The identified microRNAs expression levels in the subgroup (RSA-primary, RSA-secondary) and control group. “*”, “**” and “***” represent *p*-value < 0.05, *p*-value < 0.01 and *p*-value < 0.001, respectively

The relative expression was detected in the RSA and control group. The results revealed that the expression of miR-182-5p and miR-2110 in the villi of the RSA group was significantly lower (0.736 ± 0.139, *p* < 0.01; 1.874 ± 0.476, *p* < 0.05) than that of control group (1.830 ± 0.480; 2.493 ± 0.582) (Fig. 5A). However, the expression of miR-320a-3p was not significantly different (3.213 ± 0.786, *p* > 0.05) from that in the villi of control (2.614 ± 0.626) (Fig. 5A). The RSA group was divided into two subgroups: primary RSA group (RSA-P), and secondary RSA group (RSA-S). Similarly, the expression of miR-182-5p and miR-2110 in the RSA-P group was significantly lower (0.550 ± 0.119, *p* < 0.001; 1.390 ± 0.425, *p* < 0.05) than that in the control group. However, the difference was not significant in RSA-S group (0.994 ± 0.280; 2.544 ± 0.967; *p* > 0.05) (Fig. 5B). The expression of miR-320a-3p was not significantly different in the RSA-P group and RSA-S group (2.051 ± 0.344; 4.822 ± 1.755; *p* > 0.05) (Fig. 5B).

### The association of microRNAs expression with clinical characteristics in the RSA group

**Fig. 6:**
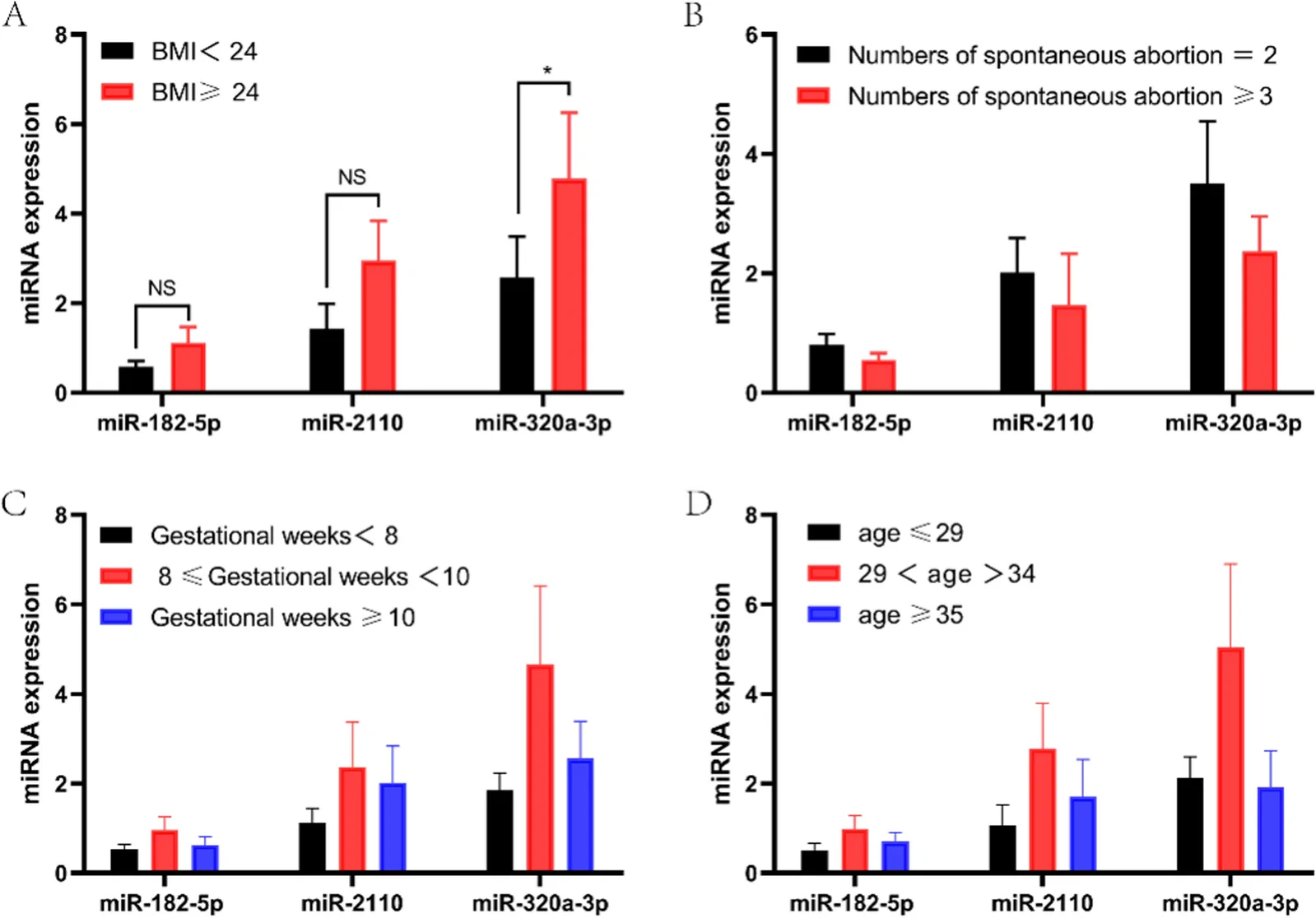
The association of microRNAs expression with Body Mass Index (BMI)(**A**), number of spontaneous abortion(**B**), gestational weeks(**C**), and age(**D**) in the RSA group

The results revealed that among all RSA patients with available BMI data, the relative expression of miR-320a-3p was significantly upregulated in those with a high BMI (9 cases) compared with those with a normal BMI (22 cases) (*p* < 0.05) (Fig. 6A). There was no significant difference in the relative expression of miR-182-5p and miR-2110 between high BMI patients and normal BMI patients (Fig. 6A), although the expression level was upregulated in high BMI patients compared with normal BMI patients. There was also no significant difference in the relative expression of miR-182-5p, miR-2110, and miR-320a-3p between twice spontaneous abortions (23 cases) and no less than 3 spontaneous abortions (8 cases) (Fig. 6B), although the expression level was downregulated in no less than three spontaneous abortions than twice spontaneous abortions. Moreover, the RSA group was categorized into three groups on the basis of gestational age: less than 8 weeks (10 cases), no less than 8 weeks and less than 10 weeks (13 cases), and no less than 10 weeks (8 cases). A greater expression level was observed in the second group, whereas there was no significant difference in the relative expression of miR-182-5p, miR-2110, and miR-320a-3p (*p* > 0.05) (Fig. 6C). The RSA patients were divided into three groups according to age: no more than 29 years old (12 cases), 30 to 34 years old (12 cases), and no less than 35 years old (7 cases), also with no significant difference observed in the relative expression of miR-182-5p, miR-2110, and miR-320a-3p (*p* > 0.05) (Fig. 6D), although a higher expression level was observed in the 30–34 years old group.

## Discussion

Numerous studies have been conducted to elucidate the etiology and pathogenesis of recurrent spontaneous abortion (RSA) and to improve its diagnosis and treatment [11–13]. Among these, research on microRNAs (miRNAs) has attracted increasing global attention, with several studies investigating differential miRNA expression in recurrent miscarriage. Ding et al. [14] reported that miR-146a-5p and miR-146b-5p were upregulated in trophoblasts treated with M1-EVs, inhibiting trophoblast migration and invasion. miR-133a and miR-101-3p in the serum-circulating system were upregulated to the greatest extent in Chlamydia trachomatis-associated RSA, and may play a role in the immunoregulation of progesterone and cytokines [15]. The present study aimed to identify dysregulated miRNAs in the villi of RSA patients, explore their correlation with clinical characteristics, and elucidate the potential mechanisms.

To exclude potential interference from chromosomal karyotype abnormalities, we compared chromosome karyotype not only with the microRNAs but also with the number of spontaneous abortions in SA patients. Our data showed that the proportion of abnormal chromosome karyotypes was significantly greater in patients with once abortion than in those with two or more abortions, suggesting that RSA is primarily driven by non‑chromosomal causes. To eliminate the confounding effect of the imbalanced karyotype distribution, we enrolled patients with two or more spontaneous abortions, namely RSA cases, as the subjects for subsequent analysis. Furthermore, no association was observed between miRNA expression levels and karyotype status, suggesting that chromosomal abnormalities do not influence miRNA expression.

In this study, 16 upregulated and 20 downregulated microRNAs were identified by miRNA sequencing. It has been reported that miR-182-5p is has been reported to be involved in colon cancer, as its overexpression directly downregulates vascular endothelial growth factor C [16]. Elevated expression of miR-182-5p in patients with preeclampsia promoted the development of preeclampsia [17]. In addition, miR-182-5p is involved mainly in inflammatory cell infiltration, goblet cell proliferation [18], angiogenesis, and lymph angiogenesis [16], and these phenotypes might play a role in RSA. In our work, miR-182-5p expression was significantly downregulated in the villus of RSA patients compared with those of healthy women. Similarly, miR-2110 was also downregulated in RSA patients. Current studies on miR-2110 have revealed an association with tumors [19–21], which are rarely involved in pregnancy or abortion. However, Castelli et al. [22] reported that miR-2110 targeted the 14-bp fragment region of the HLA-G 3’ untranslated region (3’ UTR). Moreover, our previous research demonstrated that HLA-G 3’ UTR single nucleotide polymorphism was related to RSA and threatened abortion. Collectively, these findings support the hypothesis that dysregulation of miR‑182‑5p and miR‑2110 in villi is closely linked to RSA. It is plausible that miR-182-5p and miR-2110 promote the occurrence of RSA by regulating target gene-mediated protein expression, which in turn affects affecting trophoblast proliferation, migration and apoptosis. Further in-depth studies are needed to confirm this hypothesis. Although miR-320a-3p expression level in human granulosa cells was negatively correlated with the good-quality embryo rate and live birth rate [23], no significant difference was observed in the expression of miR-320a-3p between RSA patients and healthy subjects.

Few studies have investigated primary versus secondary RSA, and the information from the current literature regarding the differences between primary and secondary RSA is inconclusive [24–27]. In order to explore the potential difference between the primary and secondary RSA group, we compared miRNA expression in both groups with that in healthy controls. Our data showed that the levels of miR-182-5p and miR-2110 decreased significantly in the primary group but not in the secondary group, whereas the level of miR-320a-3p did not differ significantly between the two groups. The updated ESHRE guideline in 2022 [27] recommended that HLA class II determination could be considered for prognostic purposes in Scandinavian women with secondary RPL after the birth of a boy. A previous study stated that primary RSA was associated with abnormal prolactin levels and was an independent risk factor for preterm delivery compared with secondary RPL [26]. These findings indicate that the etiology and pathogenesis of primary and secondary diseases are different. Taken together, miR-182-5p and miR-2110 hold promise as potential biomarkers for RSA or primary RSA, although further validation in larger cohorts is warranted.”.

The associations RSA of with clinical characteristics: maternal age, history miscarriages, gestational weeks, and BMI, are supported by a few pieces of literature [28–30]. There was no significant difference between miR-182-5p or miR-2110 expression levels and clinical characteristics in our work. Notable, miR-320a-3p expression level was significantly correlated with a higher BMI.

A previous study suggested that miRNAs (e.g., miR-199a- 5p, miR-449a) are present significantly greater levels in endometrial cancer patients with elevated BMI [31]. Obesity (elevated BMI) is known to contribute to RSA through multiple mechanisms, including dysregulated secretion of adipokines such as leptin [32]. Leptin may serve as a complementary marker of adiposity for assessment of obesity and reproductive outcomes [33], and maternal leptin (LEP) and leptin receptor (LEPR) gene polymorphisms are associated with RSA [34]. It is widely recognized that genetic polymorphisms can influence both the processing and maturation of miRNAs and the binding efficiency between miRNAs and their target genes. A recent study on lipodystrophy demonstrated that circulating miR-320a-3p levels exhibited a significant inverse relationship with plasma leptin, reflecting white adipose tissue mass and function [35]. Collectively, miR-320a-3p may be actively involved in the adipose tissue-derived signaling network that influences fertility. Notably, miR-320a-3p has been implicated as a potential negative indicator to predict good-quality embryos and live births in patients undergoing in vitro fertilization [23], further supporting its potential relevance to reproductive outcomes. Together with our observation that miR-320a-3p correlates with BMI but does not differ significantly between RSA patients and healthy controls, these data support the hypothesis that miR-320a-3p may indirectly affect reproductive outcomes by participating in obesity-related metabolic pathways, rather than acting as a direct disease-specific marker for RSA. Admittedly, the precise mechanisms underlying these findings warrant further investigation. Our future work will involve expanding the study cohort to confirm these findings and conducting mechanistic studies to elucidate the role of miR-320a-3p in the crosstalk between adiposity and RSA.

To the best of our knowledge, this is the first study to identify an association of miR-182-5p and miR-2110 with RSA, particularly primary RSA. Moreover, miR-320a-3p may be indirectly involved in RSA through its association with BMI, rather than being directly associated with the disease itself. Several limitations of this study should be acknowledged. First, the sample size was relatively small, which may limit the generalizability of our findings. Second, the current study was performed using villous tissues collected at the time of miscarriage, which only reflects the fetal side of the maternal-fetal interface and fails to capture dynamic alterations in maternal parameters during gestation. Additionally, whether the effect of miR‑320a‑3p on RSA is mediated by BMI remains to be fully elucidated. Further studies with larger, multi-center cohorts and longitudinal maternal sampling are needed to assess the clinical utility of miRNAs.

In conclusion, our study provides novel insights for the clinical management of RSA patients and lays a foundation for the development of innovative diagnostic and therapeutic strategies for RSA.

## Conclusions

In the present study, we demonstrate that miR-182-5p and miR-2110 are associated with RSA, particularly primary RSA, and that miR-320a-3p is indirectly involved in RSA through its association with BMI. These findings suggest that miR-182-5p and miR-2110 may serve as potential biomarkers for RSA and could help distinguish primary from secondary RSA, whereas miR-320a-3p may be indicative of obesity-associated metabolic status. In conclusion, miRNAs may play a role in the etiology, pathogenesis, and prognosis of RSA.

## Data Availability

The raw sequencing data generated during the current study are available in the Genome Sequence Archive for Human (GSA-Human), accession number HRA019819.
